# Linkages Between Indigenous Cultural Generativity and Sobriety to Promote Successful Aging Among Alaska Natives

**DOI:** 10.1093/geroni/igab046.829

**Published:** 2021-12-17

**Authors:** Jordan Lewis

**Affiliations:** Memory Keepers Medical Discovery Team/University of Minnesota Medical School, Duluth campus, Duluth, Minnesota, United States

## Abstract

This article builds on the People Awakening Project, which explored an AlaskaNative understanding of the recovery process from alcohol use disorder and sobriety. The presentation will explore motivating and maintenance factors for sobriety among older AN adult participants (age 50+) from across Alaska. Ten life history narratives of Alaska Native older adults, representing Alutiiq, Athabascan, Tlingit, Yup’ik/Cup’ik Eskimos, from the PA sample were explored using thematic analysis. AN older adults are motivated to abstain from, or to quit drinking alcohol through spirituality, family influence, role socialization and others’ role modeling, and a desire to engage in indigenous cultural generative activities with their family and community. A desire to pass on their accumulated wisdom to a younger generation through engagement and sharing of culturally grounded activities and values, or indigenous cultural generativity, is a central unifying motivational and maintenance factor for sobriety. The implications of this research indicates that family, role expectations and socialization, desire for community and culture engagement, and spirituality are central features to both Alaska Native Elders’ understanding of sobriety, and more broadly, to their successful aging. Sobriety can put older Alaska Native adults on a pathway to successful aging, in positions to serve as role models for their family and community, where they are provided opportunities to engage in meaningful indigenous cultural generative acts.

